# Photobiomodulation Strengthens Muscles via Its Dual Functions in Gut Microbiota

**DOI:** 10.1002/advs.202511582

**Published:** 2025-09-23

**Authors:** Prabhat Upadhyay, Asmita Banstola, Brijesh Bhayana, Mei X. Wu

**Affiliations:** ^1^ Wellman Center for Photomedicine Massachusetts General Hospital Department of Dermatology Harvard Medical School 50 Blossom Street Boston MA 02114 USA

**Keywords:** anaerobic bacteria, intense exercise, microbiota, mitochondria biogenesis, photobiomodulation

## Abstract

Photobiomodulation therapy (PBMT) alleviates fatigue and enhances mitochondrial bioenergetics, yet effects are modest when applied only to the thighs. Here, it is demonstrated that brief, non‐invasive abdominal PBMT with 980 nm light (0.1 J cm^−2^) substantially improves endurance by preserving gut epithelial integrity and modulating microbiota. In graded treadmill protocols under anaerobic stress, combined abdomen‐and‐legs PBMT nearly doubles time to exhaustion versus sham by day 20 (70.23 vs 32.38 min; *P* < 0.0001) and remains superior at day 30 (55.56 vs 32.38 min; *P* < 0.0001). PBMT preserves mitochondrial cristae and mass in gastrocnemius and soleus muscles (*P* ≤ 0.0018), achieving a fourfold increase over sham (*P* < 0.0001). Abdominal PBMT maintains seven major gut phyla disrupted by intense exercise, enriches SCFA‐producing taxa, reduces pathobionts, and elevates circulating and fecal butyrate, spermidine, L‐carnitine, and acetylL‐carnitine (all *P* ≤ 0.0001). *Ex vivo* assays under anaerobic, acidic conditions confirm PBMT‐driven expansion of key anaerobes (*P* < 0.05–0.0001). In a lactic‐acidosis model, abdominal PBMT reduces peak weight loss (≈5% vs ≈10%) and accelerates recovery. These findings establish abdominal PBMT as a non‐invasive modality that reverses epithelial injury and dysbiosis, revealing translational potential for enhancing performance, rehabilitation, and managing disorders associated with dysbiosis or mitochondrial dysfunction.

## Graphical abstract

Non‐invasive photobiomodulation therapy (PBMT) enhances muscle endurance by rewiring the gut‐muscle axis. PBMT preserves gut epithelial integrity and enriches beneficial anaerobes, driving metabolites such as butyrate, spermidine, and L‐carnitine that supercharge mitochondrial energy in skeletal muscles. This work uncovers a novel gut‐muscle communication mechanism and positions PBMT as a promising non‐invasive strategy to boost systemic metabolism and resilience.

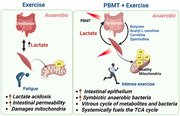

## 1 Introduction

High‐intensity exercise imposes profound metabolic stress, driving peripheral fatigue when ATP demand exceeds supply. Under limited oxygen, pyruvate is diverted to lactate, hydrogen ions accumulate, and mitochondrial efficiency deteriorates, impairing muscle contractile performance.^[^ ^1^ ^]^ While training and nutrition partially mitigate these constraints, interventions that sustain energy flux and constrain toxic metabolite buildup remain needed.^[^ ^2^ ^]^ The gut–muscle axis has emerged as a critical modulator of exercise capacity. Microbial diversity was found to correlate with muscle mass and quality; while short‐chain fatty acids (SCFAs), particularly butyrate, fuel colonocytes, maintain epithelial integrity, and influence host metabolism and inflammation.^[^ ^3^ ^]^ Conversely, exhaustive exercise can induce dysbiosis, leaky gut, and systemic inflammation, collectively impairing muscle function. This insight has motivated nutritional interventions, such as probiotics, to target muscle mass and function.^[^ ^4^ ^]^ Probiotics, comprising beneficial microorganisms consumed in adequate quantities, have shown promise in enhancing muscle mass and function through various cellular pathways. For instance, *Lacticaseibacillus casei* (LC122) and *Bifidobacterium longum* (BL986) have been found to increase skeletal muscle mass and grip strength.^[^ ^5^ ^]^ However, these approaches rely on engraftment of exogenous bacterial strains that often fail to grow in pathological or stressed colonic environments, limiting their long‐term effects, as dysbiosis eventually prevails in the stressed colon.^[^ ^6^ ^]^ Some probiotic strains may even exacerbate symptoms such as bloating, discomfort, or inflammation, requiring cautious application and close monitoring by healthcare professionals.

A modality that can restore healthy gut niches and promote the growth of beneficial anaerobes independently and synergistically would address both sides of this dyad. Photobiomodulation therapy (PBMT), also known as low‐level light therapy (LLLT), has gained attention as a therapeutic avenue for mitigating exercise‐induced physiological stress. PBMT uses specific light wavelengths, mainly in the near‐infrared range, to stimulate cellular processes and facilitate tissue repair. Its efficacy has been demonstrated in wound healing, pain management, and inflammation control.^[^ ^7^ ^]^ In addition, PBMT shows promise in enhancing glucose uptake, maintaining oxidative phosphorylation, and reducing lactate production under hypoxic conditions.^[^ ^8^ ^]^ Its potential to modulate gut microbiota was first introduced by Liebert in 2019 under the term “photobiomics”. By modulating microbiome, metabolome, and inflammatory pathways, PBMT could benefit neurological disorders such as Alzheimer's and Parkinson's diseases, where therapeutic effects were linked to specific microbial taxa like *Rikenella*.^[^ ^9^ ^]^ Yet, these benefits were largely attributed to PBMT's ability to enhance mitochondrial function and prevent apoptosis of gut epithelial cells, thereby preserving gut barrier integrity and indirectly exerting its probiotic effects.^[^ ^10^ ^]^

We hypothesized that PBMT exerted dual actions: protecting and renewing the gut epithelium while directly promoting anaerobic probiotics. The two functions operated independently yet synergistically, creating a virtuous cycle that restored host‐microbiota symbiosis and improved systemic performance. While PBMT's epithelial protective effects have been reported,^[^ ^11^ ^]^ its direct impact on gut microbiota remains underexploited until our recent findings.^[^ ^12^ ^]^ In the current study, we show that non‐invasive abdominal PBMT prior to intense exercise significantly enhanced muscle strength, with thigh illumination contributing modest effects, while the combination yielded even greater effects. The local PBMT giving rise to systemic improvements was associated with increased production of metabolites, such as L‐carnitine, acetyl‐L‐carnitine, butyrate, and spermidine, driven by enrichment of anaerobic gut bacteria. These metabolites increased mitochondrial density with healthier cristae in leg muscles, enhancing bioenergetics and resilience. These findings confirm our hypothesis and offer novel insights into the therapeutic potential of PBMT as a promising strategy to concurrently safeguard gut epithelium integrity and foster microbial homeostasis under conditions of dysbiosis and metabolic stress.

## 2 Result

### 2.1 Abdominal Illumination Strengthens Physical Endurance against Fatigue

**Figure 1:**
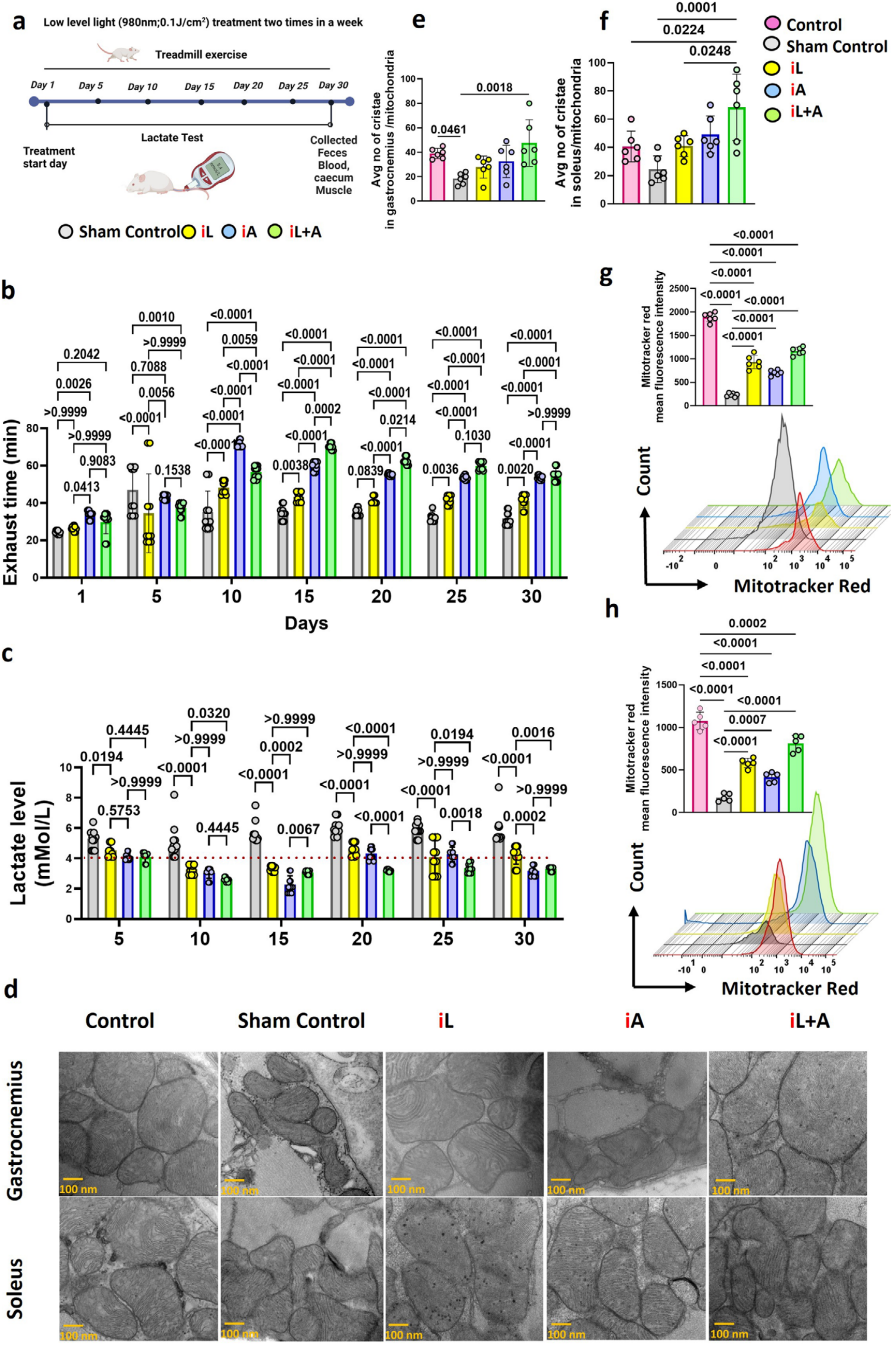
LLL illumination of the abdomen enhances endurance against fatigue caused by anaerobic exercise. a). Depiction of the frequencies and timeline of treadmill exercise and LLL illumination. b). Exhaust time is recorded on the digital display screen of the treadmill during anaerobic high‐intensity exercise. c). Lactate level (mMol L^−1^) measured during the high‐intensity exercise in anaerobic conditions. A dotted line indicates the normal level≈4.5 mMol L^−1^. d). Representative electron micrographs of gastrocnemius & Soleus muscles at 100 nm. e, f). Average number of cristae measured at 100 nm of the gastrocnemius or soleus muscles by Image J software from the electron micrograph. g, h). MitroTracker Red CMXRos staining and the mean fluorescence intensity (MFI) of stained mitochondria in gastrocnemius and soleus muscles. One representative result of two independent experiments is shown using mice at 6–8 weeks of age. The error bar indicates the SEM, *n* = 12 for b, c, 6 for e–h, and 3 for (d). *P*‐value was obtained with one‐way ANOVA and two‐way ANOVA using Bonferroni post‐test.

To determine the potential of LLL‐mediated probiotics in strengthening exercise endurance, LLL was administered to the hind limb (iL), abdomen (iA), or both (iL+A) before the mice undertook high‐intensity exercise on the treadmill under anaerobic conditions with a gradual increase in speed at an incline position (Table 2). We previously showed that 0.1 J cm^−2^ of 980 nm laser enhanced muscle cell differentiation under hypoxic conditions at levels comparable to 3 J cm^−2^ of 810 nm, representing a 30‐fold higher sensitivity of 980 nm compared to 810 nm light.^[^ ^8^ ^]^ Based on these findings, a 980 nm laser at an influence of 0.1J cm^−2^ in the cellular level was selected for LLLT. To achieve the light energy level in the mid‐depth of the mouse hind limb or abdomen tissue, light penetration was measured through hair‐shaved tissue, with a depth of 4–6 mm cut till gastrocnemius & soleus muscles (**Table** **1**). We found that light absorption was 99.9% in the presence of hair and 98.9% after shaving. However, absorption decreased to 33.3% at a depth of 4–6 mm beneath the upper layer, reaching the underlying soleus and gastrocnemius muscle tissues. Likewise, the abdomen showed 99.9% absorbance before and 98% after shaving, and 13.5% in the open abdomen. The measurement concluded that 60‐s non‐invasive illumination on the shaved hind limb with the power 170 mW was required to deliver 0.1 J cm^−2^ to the gastrocnemius & soleus muscles, while 25‐s illumination was necessary for the abdomen (Table 1). Apart from light fluence, lactate levels higher than 5 mMol L^−1^ during exercise serve as a marker of anaerobic conditions and fatigue or lactic acidosis.^[^ ^13^ ^]^ We set the running parameter per the given **Table** **2**. The lactate levels increased from 2.5 to 9 mMoL L^−1^ during 35 min of treadmill exercise with a gradual speed increment at a 10° angle incline, simulating anaerobic conditions (Figure S1a, Supporting Information). Ten treatment sessions were administered to each mouse twice weekly over 30 days, prior to treadmill exercise (**Figure** **1**a). On day 1, the mean exhaustion time increased slightly from 24.24 min in the sham treatment group to 26.47 min in the iL group receiving illumination on two hind limbs only, but this difference was not statistically significant (>0.999, Figure 1b). A significant improvement in running time was observed only with abdomen illumination (iA) (*P* = 0.0026), either alone or in combination with leg illumination (iL+A) (Figure 1b). This result was unexpected, as current practices primarily expose leg muscles only to LLL to enhance exercise performance in experimental models and clinical settings.

**Table 1:** Dose optimization of LLL on the mouse hind limb and abdomen.

**Parameters**	**Hind Limb**		
**Thigh**	**With hair**	**Without hair**	**Without hair, 4–6 mm depth**
	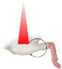	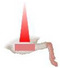	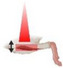
**Power applied [mW]**	170	170	170
**Power penetration [mW]**	0.17	1.87	113.4
**Power absorbed [%]**	99.99	98.9	33.3
**Calculated time (Sec) as required for 0.1 J cm** ^−^ **^2^**	∼664	∼60	∼1 sec

**Abdomen**	**With hair**	**Without hair**	**Without hair and open**
	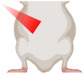	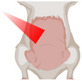	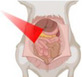
**Power applied [mW]**	220	220	220
**Power penetration[mW]**	0.22	4.4	190
**Power absorbed [%]**	99.9	98	13.5
**Calculated time (Sec) as required for 0.1 J cm** ^−^ **^2^**	∼513	∼25	∼0.59

**Table 2:** Treadmill setup for the intense anaerobic exercise.

Anaerobic Treadmill Condition (10^0^)	
Speed	Time [min]
Acclimatize	0–2 min
5 m	3–5 min
15 m	6–15 min
20 m	16–30 min
25 m	31 min‐till Exhaust

The benefits of iA treatment were evident throughout the study period and became even more pronounced during prolonged exercise, as demonstrated on days 5 and 10. By day 30, the exhaust time had significantly increased to 40.46 min in the iL group compared to 32.38 min in the sham treatment group (*P* = 0.0020, Figure 1b). The improvements were greater in the iA (52.69 min) and iL+A (55.56 min) groups (*P* < 0.0001, Figure 1b). On days 15 and 20, the iA and iL+A groups demonstrated the highest run times, reaching 59.30 and 55.20 min for iA, and 70.23 and 62.45 min for iL+A, respectively. Notably, after two weeks of illumination, the iA group exhibited more than 50% longer exhausting time than the iL group (*P* < 0.0001), while the iL+A group nearly doubled the running time (*P* < 0.0001, Figure 1b). These improvements were accompanied by significant reductions in lactate levels across the LLLT groups after two weeks of training (*P* < 0.0001, Figure 1c). The sham treatment group showed lactate levels of 5.8 mMol L^−1^, whereas the LLLT groups exhibited lower levels: 4.1 mMol L^−1^ in the iL group, 3.9 mMol L^−1^ in the iA group, and 3.2 mMol L^−1^ in the iL+A group, all below the normal range indicated by the dotted line (Figure 1c).

In anaerobic conditions, pyruvate is primarily converted into lactate owing to a lack of oxygen to support oxidative phosphorylation. While converting pyruvate to lactate allows glycolysis to continue producing ATP, prolonged anaerobic conditions can accumulate lactate and hydrogen ions, contributing to muscle fatigue and decreased exercise performance, in part ascribed to mitochondrial dysfunction in the muscles.^[^ ^14^ ^]^ We previously showed that LLL enhanced mitochondrial quantity and quality in muscle cells under anaerobic conditions.^[^ ^8^ ^]^ Similar effects on mitochondrial mass were also found in various cells and tissues under anaerobic conditions in vivo and in vitro. The PBMT‐mediated improvement of mitochondrial quantity and quality, in alignment with the findings that LLLT enhanced exhaustion time and reduced blood lactate levels under anaerobic conditions, motivated us to examine muscle mitochondrial functions. The gastrocnemius and soleus muscles contain muscle fibers, including fast‐twitch (type II) and slow‐twitch (type I) fibers. Fast‐twitch fibers are more involved in high‐intensity, explosive movements and rely heavily on anaerobic metabolism.^[^ ^15^ ^]^ After 30 days of physical endurance activities of mice over a treadmill daily, the muscles from the iL+A group exhibited a significantly higher number of healthy cristae compared to the sham treatment controls, as revealed by transmission electron microscopy (TEM) (Figure 1d). Quantitative measurements revealed a statistically significant increase in healthy cristae in the iL+A group compared to the sham treatment controls (*P* = 0.0018 for gastrocnemius; *P* = 0.0001 for soleus) (Figure 1e,f). The iL or iA alone regimen also exhibited increased healthy cristae compared to the sham treatment control, reaching the levels seen in non‐exercised controls. The combined iL+A regimen promoted mitochondrial biogenesis and preserved cristae morphology, as evidenced by the higher number of healthy cristae, potentially fueling ATP production and improving electron transport chain (ETC) efficiency during intense exercise.

Mitochondrial mass in contralateral hind limb muscle tissues was next analyzed by flow cytometry (Figure 1g,h). The extensive exercise substantially reduced mitochondrial mass (6–8‐fold, *P* < 0.0001) in gastrocnemius and soleus muscles compared to the non‐exercised controls, consistent with exercise‐induced muscle injury. The iL+A treatment significantly mitigated this loss, increasing mitochondrial mass by 4‐fold over the sham treatment control (*P* < 0.0001) and 1.4‐fold compared to the iL group, though still lower than non‐exercised controls. The iL or iA alone treatment also significantly increased mitochondrial mass (3–4‐fold, *P* < 0.0001 in both; *P* < 0.0001 & *P* = 0.0007 for soleus and gastrocnemius, respectively) relative to the sham treatment control, although their effects were slightly less pronounced than the iL+A regimen. Despite the increased mitochondrial mass, the iL‐treated mice showed no improvement in exhaustion time until day 15, with performance gains stabilizing from days 25 to 30 (Figure 1b). This suggests that local treatment benefit may be transient, a limitation commonly observed in clinical applications. These findings highlight the potential of LLLT for enhancing physical endurance during intense exercise, with iL+A proving more effective than muscle‐targeted illumination alone.

### 2.2 Abdominal Illumination Strengthens the Symbiotic Interplay among Gut Microbiota, Metabolites, and Epithelial Cells

**Figure 2:**
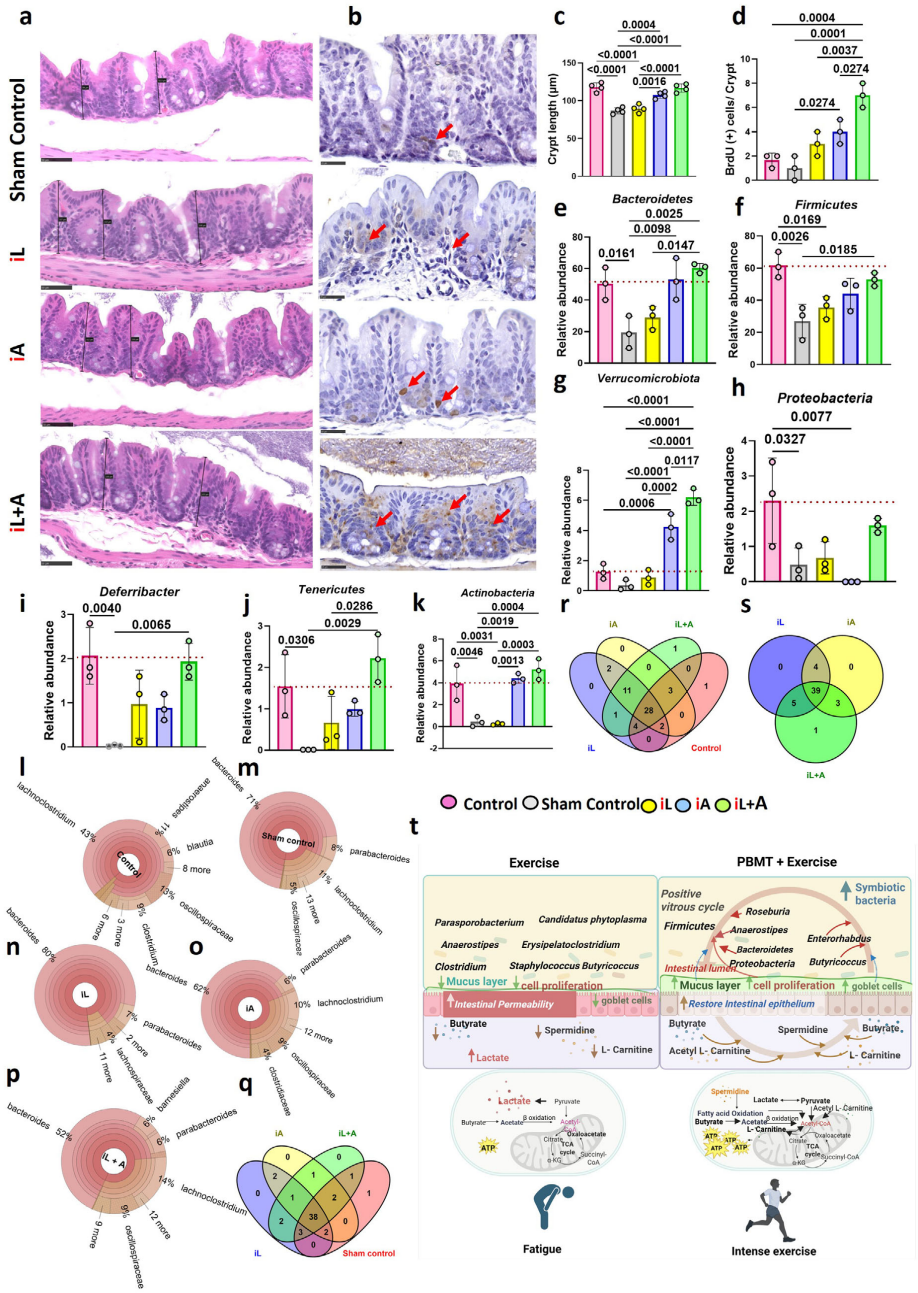
Abdominal illumination sustains the gut epithelial integrity and microbiota against exercise‐mediated dysbiosis. Mice were exercised and treated as depicted in Figure 1a. Tissues and feces DNA were collected at the end of the study (30 days). a, b). H&E (a) and BrdU (b) staining of the caecum after exercise for 30 days with or without the indicated treatments. Red arrows indicate the BrdU‐positive cells in the crypt. Scale bars, 50 µm for (a) and 25 µm for (b). c). Comparison of crypt lengths of the caecum measured through image viewing NDP view 2 software (n = 10). d) Comparison of BrdU‐positive cells per crypt of the caecum (n = 10). e–k). The relative abundance of major phyla identified from feces DNA using the qPCR includes *Bacteriodetes* (e), *Firmicutes* (f), *Verrucomicrobiota* (g), *Proteobacteria* (h), *Deferribacter (i), Tenericutes* (j), and *Actinobacteria (k)* (*n* = 6). The error bar indicates the SEM. *P* value obtained by one‐way ANOVA with Bonferroni post‐test. Korona plots depicting gut microbiota composition across experimental groups. The plots illustrate the relative abundance and diversity of microbial taxa in Control (l), Sham Control (m), iL (n), iA (o), and iL+A (p) groups. q–s). Venn diagrams of the taxonomy files are plotted to compare the number of unique and common bacterial species in the exercise and non‐exercise control groups. t). A schematic summary illustrates the reversal of a vicious cycle (left) to a virtuous cycle (right) by PBMT, driven by improved metabolites and interactions between gut epithelial integrity and microbiota. In contrast, the exercise group shows fatigue, associated with elevated lactate levels and an increase in pathogenic bacteria.

Aerobic exercise has been reported to increase microbiota biodiversity and promote beneficial metabolic function.^[^ ^16^ ^]^ In contrast, exhaustive exercise training often induces gut dysbiosis, resulting in adverse metabolic effects and systemic inflammation.^[^ ^17^ ^]^ The ability of abdominal PBMT to improve physical endurance is likely attributed to its beneficial effects on gut microbiota and epithelial integrity. As shown in **Figures** **2**a and S1b (Supporting Information), the sham‐treated controls exhibited an inflamed caecum with disrupted crypt architecture, whereas mice treated with the iA and iL+A regimens demonstrated significantly improved crypt architecture, closely resembling the non‐exercised control as measured by crypt length (*P* = 0.0004 & *P* < 0.0001, Figure 2a,c; Figure S1b, Supporting Information). Additionally, bromodeoxyuridine (BrdU) staining of the same caecum section revealed a higher number of BrdU‐positive cells, indicative of increased epithelial cell proliferation,^[^ ^18^ ^]^ in the iA and iL+A groups, with the latter showing significantly stronger effects than the former (*P* = 0.0274 & *P* = 0.0001, Figure 2b,d; Figure S1c, Supporting Information). Intense physical exercise can often alter the host's physiological environment, including a decrease in gut pH caused by elevated lactate production and increased intestinal permeability (“leaky gut”). These changes directly affect the abundance and diversity of anaerobic bacteria due to oxygen leakage and elevated production of reactive oxygen species (ROS).^[^ ^17^ ^]^ PBMT can prevent apoptosis of gut epithelial cells, as previously demonstrated,^[^ ^12^ ^]^ and/or by increasing epithelial cell proliferation (Figure 2b,d; Figure S1c, Supporting Information). While this approach does not specifically quantify tight junction integrity or epithelial survival markers, it provides supportive evidence that PBMT may promote epithelial renewal and contribute to overall gut lining health.

As for the effects of LLL on microbiota, qPCR analysis of fecal 16s rRNA genes revealed severe declines in the abundance of all seven major phyla tested in the sham‐treated group, including *Bacteroidetes*, *Firmicutes*, *Verrucomicrobia*, *Proteobacteria*, *Deferribacteres*, *Tenericutes*, *and Actinobacteria* as compared to the non‐exercise controls, highlighted by a dashed line (Figure 2e–k). These observations verified the disruption of gut microbial populations by intense exercise and validated the relevance of the exercise model. Interestingly, the iA treatment effectively preserved the abundance and diversity of all tested gut phyla to normal levels, with further improvement observed in the iL+A regimen (Figure 2e,f,i–k), with the exceptions of *Verrucomicrobia* (Figure 2g) and *Proteobacteria* (Figure 2h). Interestingly, the phylum *Verrucomicobiota* and *Tenericutes* showed an abundance exceeding the normal range in the iA and iL+A group. These phyla significantly impact gut microbiota and muscle improvement during anaerobic exercise. *Verrucomicrobiota*, particularly the genus *Akkermansia*, is associated with improved metabolic health and enhanced exercise performance. *Tenericutes*, albeit to a lesser extent, may play a role in energy metabolism and muscle function during intense physical activity. This phylum, which includes the genus *Mycoplasma*, has been associated with various metabolic processes and may contribute to the body's adaptation to anaerobic exercise.^[^ ^19^, ^20^ ^]^ Some studies suggest that *Tenericutes* could regulate glucose metabolism and muscle tissue repair, potentially enhancing recovery and performance during high‐intensity workouts.^[^ ^21^, ^22^ ^]^

To analyze the diversity at the genus or species level affected by LLL, the 16S rRNA gene analysis of fecal DNA validated the significant improvement in gut microbiota diversity in mice undergoing high‐intensity exercise in anaerobic conditions with the treatment regimens of the iA or iL+A regimens. A total of 689 operational taxonomic units (OTUs) were identified by Illumina platform sequencing (Figure 2l–p). The Korona plots illustrate the gut microbiota composition across five experimental groups: Control, sham control, iL, iA, and iL+A. A clear shift in microbial diversity is observed among these groups. The sham control group displayed reduced diversity, with ≈90% of the microbiota dominated by a few taxa and only ≈10% representing minor microbes (Figure 2m). In comparison, the control group maintained a slightly better profile with ≈85% dominant and ≈15% minor taxa (Figure 2l). The iL group showed moderate diversity improvement, with ≈75% dominant and ≈25% minor taxa (Figure 2n). Notably, the iA group further increased microbial evenness, with ≈70% dominant and ≈30% minor taxa (Figure 2o). The iL+A group, which received both light exposure and exercise, demonstrated the highest diversity, with only ≈65% dominance and ≈35% representation of minor taxa (Figure 2p). These results, as depicted in the Korona plots, highlight the iL+A group as having the most balanced and diverse microbiota, suggesting a synergistic enhancement from the combined interventions over individual treatments and the sham control. The Venn diagram showed the common richness of 38 genera in all exercise groups irrespective of the treatments (Figure 2q), 28 between non‐exercise control & different illuminations (Figure 2r), and 39 in all illumination groups regardless of whether iL, iA, and combined iA and iL were administered (Figure 2s). Among all groups, the focus was on anaerobic bacterial genera categorized into obligate and facultative types, spanning the major phyla *Bacteroidota, Firmicutes, Tenericutes*, and *Verrucomicrobiota*, given their significant abundance observed in LLL‐treated groups (Figure 2e–k). The gastrointestinal tract harbors a diverse bacterial community, with *Firmicutes* dominating ≈50–60% of the total microbial population, particularly in anaerobic regions such as the distal small intestine and colon.^[^ ^23^ ^]^ Among all groups (Figure S2a–e, Supporting Information), we screened for obligate and facultative anaerobic bacteria, especially in the context of muscle mitochondria and exercise. Key anaerobic genera within *Firmicutes* (Figure S2f, Supporting Information)‐including *Oscillospira, Ruminococcus, Blautia, Dorea, Anaerotruncus*, and *Lactobacillus*‐play crucial roles in SCFA production, particularly butyrate, which is essential for mitochondrial function and muscle energy metabolism.^[^ ^24^, ^25^, ^26^, ^27^ ^]^ Notably, *Oscillospira* was enriched in the iL+A group (9.3%) compared to sham controls (4.5%). The iL+A group exhibited a more favorable gut microbiota profile for muscle function and mitochondrial efficiency, with increased levels of *Oscillospira* (9.3%) and *Ruminococcus* (1.4%) (Figure S2f, Supporting Information), both well‐known SCFA producers supporting gut‐muscle crosstalk and anaerobic energy metabolism.^[^ ^28^ ^]^ Among these, *Ruminococcus*, which was highest in the control group (2.4%), plays a critical role in gut barrier integrity and energy production, reinforcing its significance in exercise adaptation.^[^ ^29^ ^]^

Beyond *Firmicutes*, notable shifts were observed in the *Bacteroidota* phylum, where *Alistipes* increased in iA (11.2%) and iL+A (13.7%) groups compared to sham (1.1%). Similarly, *Blautia*, an SCFA producer, was more abundant in non‐exercise controls (13.6%) but significantly reduced with iL+A treatment (9.0%), suggesting microbial adaptation under PBMT intervention.^[^ ^30^ ^]^ Furthermore, the iL+A group displayed significant enrichment of beneficial anaerobes, including *Filifactor* (6.6%), compared to sham (0.09%), consistent with enhanced microbiota‐driven metabolic adaptation. Other enriched beneficial genera included *Erysipelatoclostridium* (2.1%), *Anaerobacterium* (5.1%), *Parasporobacterium* (4.2%), and *Papillibacter* (8.3%) (Figure S2f, Supporting Information), all of which support mitochondrial efficiency and energy homeostasis.^[^ ^31^ ^]^

In contrast, the sham control groups exhibited a significantly higher abundance of potentially pathogenic bacteria, particularly *Parabacteroides* (74.2%)(Figure S2f, Supporting Information), which is associated with gut inflammation, metabolic dysregulation, and impaired gut barrier function.^[^ ^32^ ^]^ PBMT intervention, especially in the iL+A group, significantly reduced these pathogenic bacteria, lowering *Parabacteroides* to 9.8%, restoring microbial balance, and improving gut health. Similarly, the iL and iA groups also showed a reduced abundance of *Parabacteroides*, at 56.9% and 21.9%, respectively (Figure S2f, Supporting Information). The iL+A group demonstrated a greater diversity of symbiotic anaerobic bacteria, closely resembling the microbial profile of non‐exercise controls. The presence of *Lactobacillus* (0.45%) underscores an improved host‐microbe symbiotic relationship, facilitating anaerobic energy metabolism, reducing inflammation, and supporting mitochondrial function during muscle activity.^[^ ^33^, ^34^ ^]^ Additionally, the iL+A group exhibited higher levels of *Lactobacillus* (0.45%) and *Lactococcus* (0.51%) compared to sham controls (*Lactobacillus*: 0.06%, *Lactococcus*: 0.11%). These facultative anaerobes play a critical role in lactate metabolism, converting excess exercise‐induced lactate into butyrate, thereby reducing muscle fatigue and optimizing ATP production^[^ ^35^, ^36^ ^]^ (Figure S2f, Supporting Information). As summarized in Figure 2t, exhaustive exercise increases intestinal permeability and disrupts gut microbiota, resulting in decreased levels of beneficial metabolites such as butyrate, spermidine, and L‐carnitine, while increasing lactate, which promotes systemic inflammation and fatigue^[^ ^37^, ^38^, ^39^, ^40^ ^]^ (left). PBMT counteracts these adverse effects by fostering symbiotic bacteria, restoring intestinal epithelial integrity, and enhancing energy metabolism (right). These protective effects form a positive microbiota cycle, enhance cell proliferation, strengthen the intestinal barrier, and promote beneficial anaerobic bacteria (*Firmicutes, Bacteroidetes, Verrucomicrobiota, and Tenericutes*). The metabolic adaptations include increased ATP production via fatty acid oxidation, reduced lactate accumulation, and enhanced butyrate synthesis, ultimately improving endurance and mitigating exercise‐induced fatigue (right).

### 2.3 Abdominal Illumination Augments Anaerobic Bacteria and Their Metabolites

**Figure 3:**
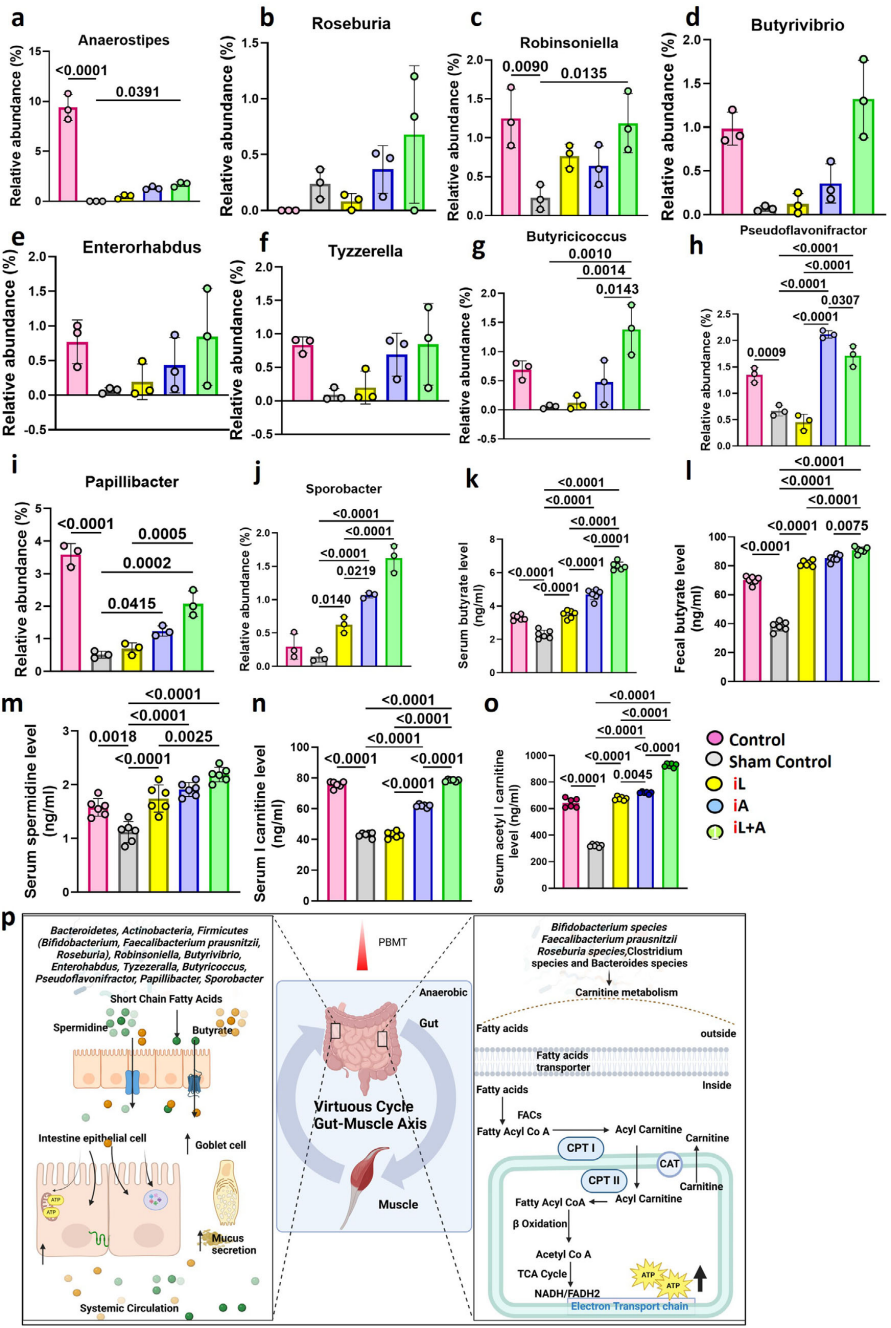
Abdominal illumination modulates the symbiotic interplay between gut microbiota and their metabolites. Mice were exercised and treated as depicted in Figure 1a. Fecal and blood samples were collected at the end of the study (30 days). Relative abundances of *Anaerostipes* (a), *Roseburia* (b), *Robinsoniella* (c), *Butyrivibrio* (d), *Enterorhabdus* (e), *Tyzzerella* (f), *Butyricoccus* (g), *Pseudoflavonifractor* (h), *Papillibacter* (i), and *Sporobacter* (j). Butyrate (ng mL^−1^) was quantified by (GC‐MS/Q‐TOF) in the serum (k) and feces (l). Bacterial metabolites were quantified in serum through LCMS/MS‐Q‐TOF positive mode, including Spermidine (m), L Carnitine (n), and Acetyl L Carnitine (o). The error bar indicates the SEM. P value obtained by one‐way ANOVA with Bonferroni post‐test. The error bar indicated the SEM, *n* = 3 for a–j and n = 6 for k–o (p). The summary illustrates the gut‐muscle axis activated by PBMT‐mediated probiosis, which enhances carnitine and short‐chain fatty acid metabolism and strengthens systemic mitochondrial ATP production during exercise.

Anaerobic bacteria predominantly colonize the large intestine, thriving in the oxygen‐depleted environments of the colon and, to a lesser extent, the cecum.^[^ ^19^ ^]^ These bacteria ferment dietary fibers that escape digestion in the small intestine, producing SCFAs as key metabolic byproducts.^[^ ^26^ ^]^ The primary SCFAs include acetate, propionate, spermidine, and butyrate, which play vital physiological roles. Butyrate, in particular, serves as the main energy source for colonic epithelial cells, contributing to gut barrier integrity and immune modulation.^[^ ^41^, ^42^ ^]^ Meanwhile, spermidine promotes autophagy in muscle cells and exhibits anti‐aging effects.^[^ ^43^ ^]^ Mice treated with the iA and iL+A regimens demonstrated significantly higher levels of spermidine (*P* < 0.0001, **Figure** **3**m) in serum and butyrate in both serum (Figure 3k) and feces (Figure 3l) compared to sham‐treated controls. The combined iL+A regimen was notably more effective than either treatment alone. Acetate and propionate were primarily derived from *Bacteroidetes* and *Actinobacteria* (e.g., *Enterorhabdus*), while butyrate was produced by *Firmicutes*, including *Anaerostipes*, *Butyrivibrio*, and *Roseburia* species. Consistent with this, mice treated with the iL+A regimen displayed a higher abundance of butyrate‐producing genera compared to sham controls, including *Anaerostipes* (*P* = 0.0391, Figure 3a), *Roseburia* (Figure 3b), *Robinsoniella* (*P* = 0.0135, Figure 3c), *Butyrivibrio*, *Enterorhabdus*, *Tyzzerella*, *Butyricicoccus* (Figure 3d–g), *Pseudoflavonifractor* (*P* < 0.0001), *Papillibacter* (*P* = 0.0002), and *Sporobacter* (*P* < 0.0001, Figure 3h–j). In the iA group, significant increases were also observed in *Pseudoflavonifractor* (*P* < 0.0001), *Papillibacter* (*P* = 0.0415), and *Sporobacter* (*P* < 0.0001), while the iL group showed significant changes in *Pseudoflavonifractor* (*P* < 0.0001) and *Sporobacter* (*P* = 0.0140). These changes in microbial abundance were reflected in the significantly elevated butyrate levels observed in both serum and feces of the LLL‐treated groups compared to sham controls.

Certain species within the *Firmicutes* phylum can convert lactate into butyrate or propionate via SCFA's metabolism, making lactate utilization an indirectly beneficial process.^[^ ^35^ ^]^ Moreover, acetylation and deacetylation, relying on acetyl groups provided by acetyl‐CoA that can be converted by SCFAs, play pivotal roles in overall metabolic and exercise performance.^[^ ^44^ ^]^ These two SCFAs or metabolites, butyrate & spermidine, are linked with the gut epithelial and mitochondrial function and play a multifaceted role in supporting the health of gut epithelial cells by providing energy, promoting cell proliferation, and exerting anti‐inflammatory effects.^[^ ^45^ ^]^ The systemic fueling of mitochondria during intense exercise shows the intricate relationship between the unique metabolites and the unidentified responsible microbiota. Apart from the metabolic byproducts produced by anaerobic bacteria, carnitine and acetyl‐L‐carnitine are synthesized through multi‐step enzymatic pathways and play essential roles in cellular energy metabolism.^[^ ^46^ ^]^ These compounds facilitate the transport of long‐chain fatty acids into the mitochondria for β‐oxidation, a process critical for efficient energy production. Specifically, carnitine, through the action of carnitine palmitoyltransferase I (CPT‐I), transfers fatty acyl‐CoA from the cytoplasm across the mitochondrial membrane. Inside the mitochondria, carnitine palmitoyltransferase II (CPT‐II) catalyzes the transfer of the fatty acyl group back to CoA, enabling β‐oxidation to break down fatty acids into acetyl‐CoA, which fuels ATP production^[^ ^47^, ^48^ ^]^ (Figure 3p). The levels of L‐carnitine (Figure 3n) and acetyl‐L‐carnitine (Figure 3o) were significantly higher (*P* < 0.0001) in all illuminated groups compared to the sham‐treated group. Notably, the dual iL+A treatment resulted in significantly greater concentrations of L‐carnitine and acetyl‐L‐carnitine in the serum compared to either treatment alone. This highlights how the process supports a positive feedback loop, systemically enhancing mitochondrial ATP production while enriching the gut microbiota during exercise.

In summary (Figure 3p), with PBMT applied to the abdomen, anaerobic bacteria (Bacteroidetes, Actinobacteria, Firmicutes, Bifidobacterium, Faecalibacterium prausnitzii, Roseburia, Tyzzerella, Butyricoccus, Papillibacter, and Sporobacter) are promoted to colonize the intestine, producing SCFAs such as butyrate and spermidine, which support gut barrier integrity. These anaerobic bacteria also contribute to increased goblet cell proliferation for cellular repair against intestinal damage. Additionally, PBMT enhances fatty acid oxidation (FAO) and mitochondrial ATP production by upregulating carnitine metabolism. L‐carnitine and acetyl‐L‐carnitine transport long‐chain fatty acids into mitochondria, where β‐oxidation generates acetyl‐CoA for the TCA cycle, fueling ATP synthesis. Notably, PBMT‐treated mice displayed significantly higher L‐carnitine and acetyl‐L‐carnitine levels, amplifying muscular endurance and systemic energy balance. PBMT fosters a “virtuous cycle” within the gut‐muscle axis, promoting SCFA synthesis, gut integrity, and mitochondrial bioenergetics. This synergy mitigates exercise‐induced fatigue, optimizing performance and recovery.

### 2.4 LLL Enhances Anaerobic Bacterial Abundance Ex Vivo

**Figure 4:**
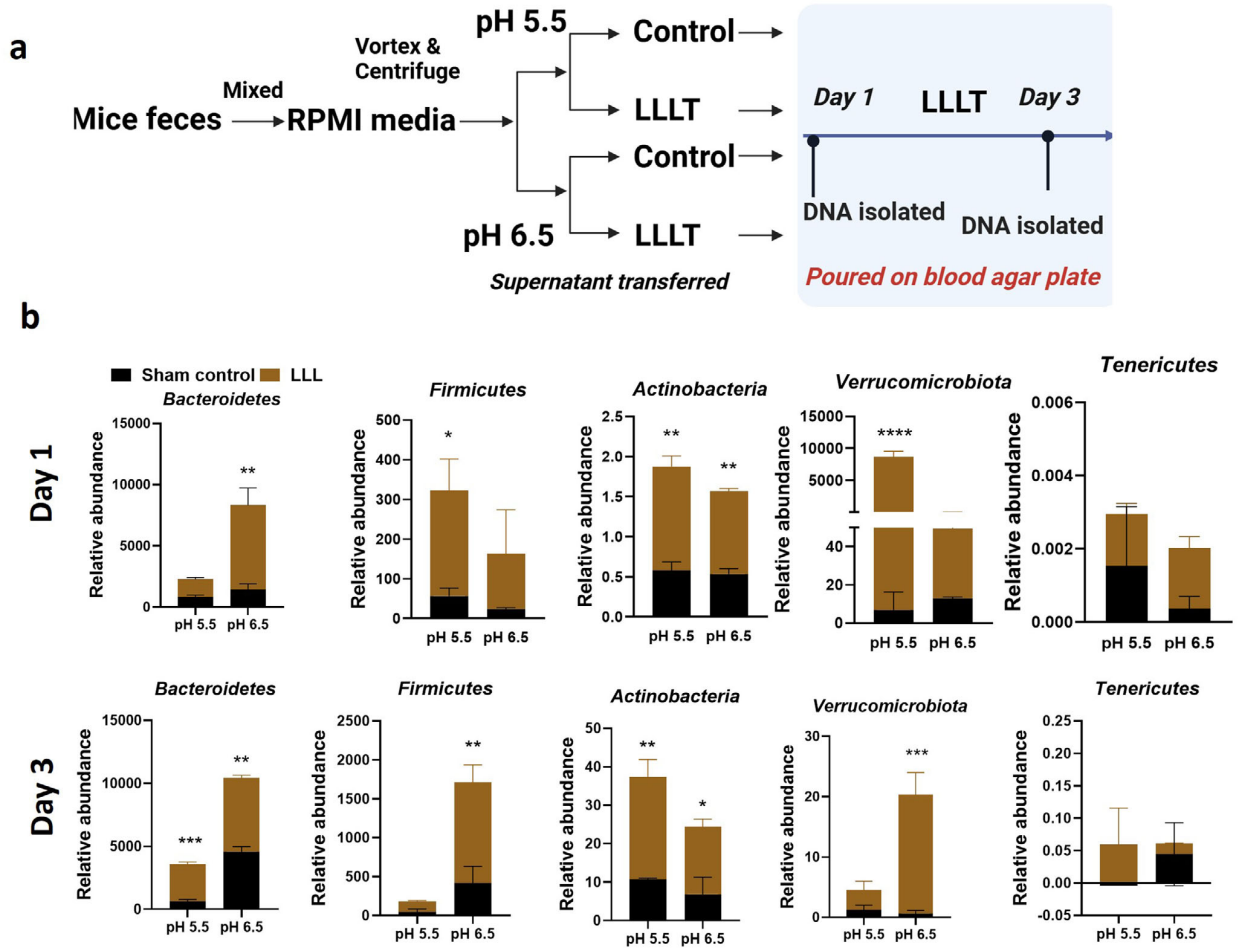
LLL promotes the growth of facultative anaerobic gut bacteria. a). A schematic diagram depicts the experimental procedure. b). The relative abundance of major phyla *Bacteriodetes, Firmicutes, Actinobacteria, Verrucomicrobiota*, and *Tenricutes* was determined by qPCR analysis of DNA prepared from bacterial cultures on days 1 and 3 after LLL or sham treatments. The experiment was repeated three times with similar results. Error bars indicate means ± SEM. Statistical analyses were performed using two‐way ANOVA with Fisher's LSD. ^*^ *p*<0.05, ^**^ *p*<0.01, ^***^ *p*<0.001, ^****^ *p*<0.0001. (*n* = 6).

Anaerobic or facultative anaerobic bacteria belonging to the *Firmicutes* phylum are particularly abundant in the colon due to anaerobic conditions with a relatively low pH (5.5–7). These bacteria ferment undigested carbohydrates, producing SCFAs crucial in maintaining cellular energy homeostasis and gut health.^[^ ^49^ ^]^ Although LLL‐mediated normalization of gut microbiota was observed, it remained debated whether LLL directly affected bacterial growth in the gut during intense exercise. Current perspectives in the field of PBM suggest that LLL primarily mitigates damage to the gut epithelium, indirectly contributing to the healthy gut microbiota. We hypothesized that LLL could directly affect the growth of anaerobic bacteria, apart from its ability to protect gut epithelial cells from apoptosis, as previously demonstrated.^[^ ^12^ ^]^ The dual functions result in a positive feedback cycle to mitigate the dysbiosis for a durable effect, in contrast to the transient effect of feeding probiotic strains. To explore the direct impact of illumination on gut bacteria enrichment under anaerobic conditions, we designed an ex vivo study, as outlined in **Figure** **4**a, mimicking the anaerobic and low pH conditions in the colon of mice undergoing intense exercise. The same major phyla examined in the in vivo study were assessed in the ex vivo study to correlate the results between in vivo and ex vivo (Figure 2e–k). Overall, LLL facilitated the growth of these anaerobic bacteria in pH 5.5, 6.5, or both, manifested with a relative abundance *on day 1 and/or day 3* in LLL‐treated cultures compared to sham‐treated ones (Figure 4b).

Specifically, *Bacteroidetes* were relatively more abundant across both pH levels and days in the LLL‐treated cultures than in the sham‐treated cultures. Despite a significant difference only at pH 6.5 on day 1 (*p* < 0.01), the difference was extended to pH 5.5 on day 3 (*p* < 0.001) (Figure 4b). This aligned with the restoration of the same phyla in mice treated with the iL+A regimen to the non‐exercise control level and more than the exercise control (Figure 2e). Similarly, *Firmicutes* exhibited significant changes on days 1 and 3, in which the relative abundance of *Firmicutes* was significantly higher (*p* < 0.05) in LLL‐treated, pH 5.5 cultures only on day 1 and became more significant as the culture was extended for 3 days at pH 6.5 (*p* < 0.01). The ability of LLL to enhance the abundance of *Firmicutes* is pivotal to increasing SCF production that helps maintain gut homeostasis by positively regulating epithelial integrity. Additionally, many facultative anaerobes within this phylum convert excess lactate into beneficial metabolites such as acetate, butyrate, and propionate.^[^ ^35^ ^]^ An increased *Firmicutes* was also seen in the mice treated with either the iL+A or iA regimen, as revealed by microbiome analysis from the feces of the mice (Figure S2f, Supporting Information). Several anaerobic *Firmicutes* bacteria, for instance, produce butyrate, a SCFA that enhances mitochondrial function and energy metabolism.^[^ ^26^ ^]^ Significant growth of *Actinobacteria* promoted by LLL was observed at both pH levels (*p* < 0.001 and *p* < 0.01) and days, with a relatively higher abundance (*p* < 0.001) at pH 5.5 (Figure 4b). Similarly, the abundance of *Verrucomicrobiota* was relatively higher in the LLL‐treated culture, showing a more pronounced increase at pH 5.5 (*p* < 0.0001) on day 1 and vice versa on day 3 (*p* < 0.001). Thus, LLL directly increased the abundance of anaerobic bacteria ex vivo, in alignment with their in vivo effects on the growth of these bacteria during anaerobic exercise.

### 2.5 Extending the Benefits of Abdominal Illumination to Dysbiosis or Acidosis

**Figure 5:**
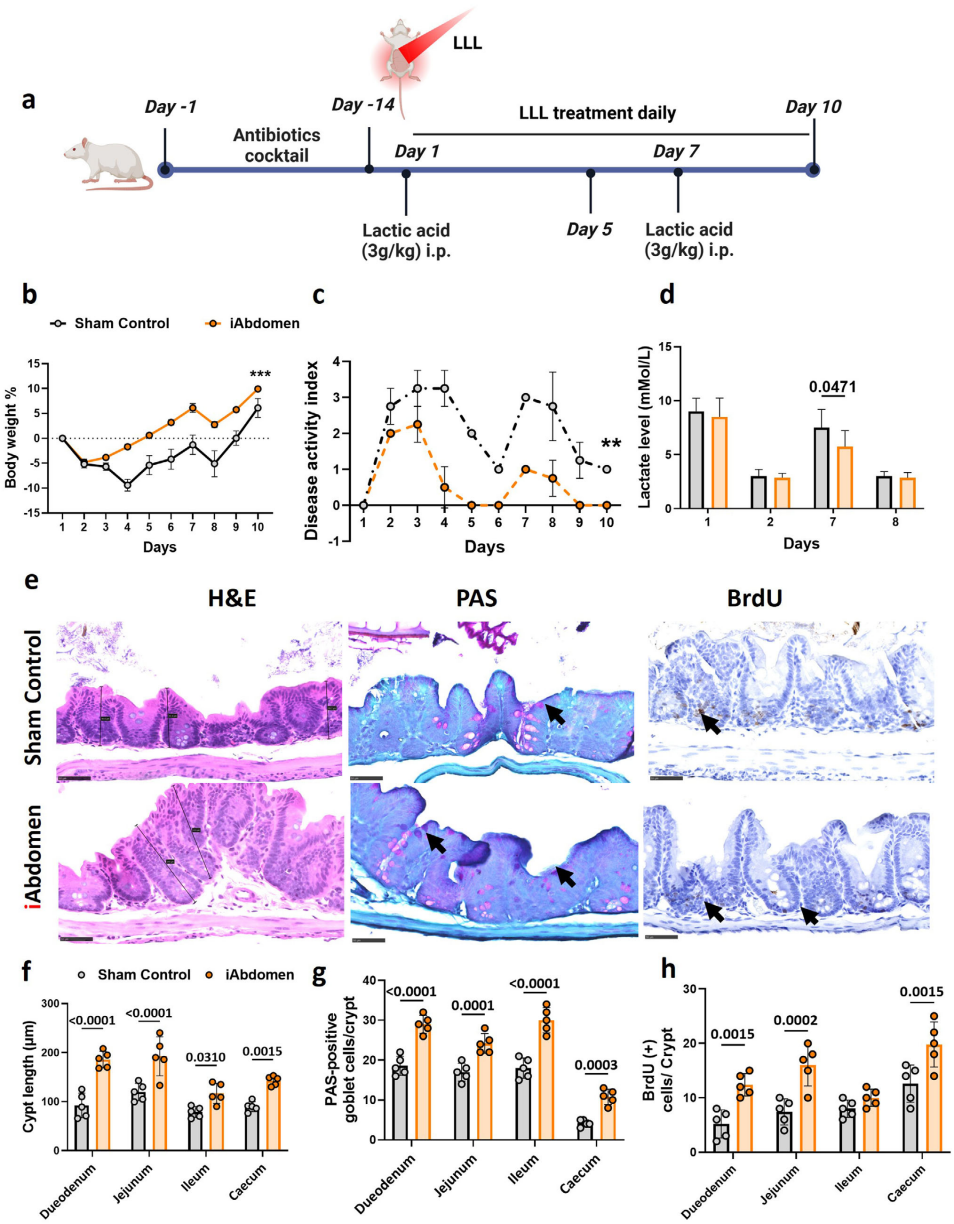
Abdominal LLLT sustains the gut epithelial integrity under lactate‐mediated acidosis. a) A schematic diagram illustrates the experimental timeline and treatments. b). Percentage changes in body weight were recorded during the experiment relative to day 1. c) The Disease Activity Index (DAI) score was assessed based on weight loss, diarrhea, and the presence of blood in the stool. Weight loss was scored as: 0, none; 1, 1–5%; 2, 5–10%; 3, 10–20%; and 4, >20%. Diarrhea was scored as: 0, normal; 2, loose stools; 3, watery diarrhea. The presence of blood in stool was scored as: 0, normal; 2, slight bleeding; and 3, gross bleeding. d). Lactate level (mMol L^−1^) was measured on the indicated days after L‐lactic acid injection. e). Representative images of the caecum after H&E, PAS, and BrdU staining on day 10 after two lactic acid injections. Scale bar, 50 µm. f). Cypt length was measured by the image viewing NDP View 2 software on the H&E‐stained sample prepared as (e). g) Positive goblet cell counts per crypt blindly after PAS staining prepared as (e). h) BrdU‐positive cell/ crypt counted after BrdU staining as (e). *n* = 10 for b–d and *n* = 6 for f–h. The error bar indicates the SEM. *P*‐value was obtained with two‐way ANOVA using the Bonferroni post‐test.

Apart from intense exercise, lactate levels are elevated during gut inflammation, such as in inflammatory bowel disease (IBD), in cancer patients, owing to the Warburg effect of cancer cells, and infections with pathogens that utilize lactate as a carbon source.^[^ ^50^ ^]^ The acidic gut environment can exacerbate dysbiosis by promoting the growth of pathogenic bacteria like *Escherichia coli* (enterotoxigenic strains), *Clostridium difficile*, and *Fusobacterium nucleatum* and by increasing the production of toxic metabolites, such as hydrogen sulfide, ammonia, and secondary bile acids, which injure the gut epithelium and induce gut leaky.^[^ ^51^ ^]^ Moreover, chronic lactic acidosis impairs mitochondrial function, driving oxidative stress and inflammation, thereby contributing to systemic diseases such as metabolic syndrome, cardiovascular disorders, and even neuroinflammation.^[^ ^52^ ^]^ To exploit LLL‐mediated probiotics in the broader implications of dysbiosis‐associated diseases, sodium L‐lactic acid was intraperitoneally administered to mice at a dosage to achieve high lactate levels in the blood without severe body loss in a pilot study. A dose of 1 and 2 g kg^−1^ induced ≈5–7mMol L^−1^ lactate, whereas a 4 g kg^−1^ dose triggered toxicity as the lactate level became excessively high at ≈20–25mMol L^−1^, resulting in the death of the mice (Figure S3a, Supporting Information). Therefore, an intermediate dose of 3 g kg^−1^ of lactic acid capable of inducing a significant lactate level of ≈9–10mMol L^−1^, as indicated with the dashed line, was selected for the study (Figure S3b, Supporting Information). First, an antibiotic cocktail was orally administered daily for 14 days to deplete the existing gut bacteria as above, followed by an intraperitoneal injection of lactic acid according to a published protocol (**Figure** **5**a). The mice suffered from severe body weight loss, reaching a maximal level of 10% loss by day 4 in the sham treatment controls, and did not recover until day 7 (Figure 5b). Under similar conditions, abdominal illumination significantly mitigated the body weight loss, showing only a 5% loss on day 2, followed by a steady recovery for 2–3 days and full recovery on day 5 (Figure 5b). Following lactic acid injection, blood lactate increased to ≈9 mMol L^−1^ on day 1, but it was normalized to ≈3–4 mMol L^−1^ on day 2 (Figure 5d). The liver plays a critical role in lactate metabolism, primarily through the Cori cycle, where lactate is converted into glucose via gluconeogenesis. Additionally, the kidneys help excrete excess lactate, while the respiratory system regulates lactate clearance by regulating blood pH through CO_2_ exhalation. As lactate accumulates, it dissociates into lactate ions and hydrogen ions, decreasing blood pH (acidosis). In response, the respiratory centers increase ventilation to expel CO_2_, helping to reduce the acidic load and stabilize the blood pH. Thus, within 24–48 h, these body's compensatory mechanisms efficiently restored normal lactate levels. However, during the lactate metabolic process, even transient increases in lactate levels can profoundly impact gut conditions due to a pH drop that increases gut permeability and induces dysbiosis and inflammation.^[^ ^53^ ^]^ These pathological alterations can be assessed using a Disease Activity Index (DAI), which incorporates parameters such as stool consistency (e.g., watery stools or diarrhea), frequency, and pH. It was found that the DAI peaked on days 2–4 and declined on day 5 with a significantly higher score in all time points in the sham treatment control than the LLL‐treated group (*p* < 0.05) (Figure 5c). Moreover, the DAI never returned to normal in the mice sham‐treated, but it was back to zero on day 5 with abdominal light exposure. To assess the mouse tolerance to lactate, we reinjected L‐lactic acid after DAI returned to zero for two days in the mice LLL‐treated. We found significantly lower lactate levels in the LLL‐treated mice (*p* = 0.047) this time (Figure 5d), with only a modest body weight loss and DAI score, followed by a rapid recovery of both (Figure 5a–c, days 7–10). In contrast, the sham treatment control showed the same lactate level. However, the loss in body weight and DAI score was not significant.

The small intestine, consisting of the duodenum, jejunum, and ileum, has a distinct environment compared to the colon. Factors such as faster transit time, greater exposure to digestive enzymes, and a more neutral to alkaline pH make it less conducive to microbial growth than the colon. However, its epithelium experiences constant renewal, whereby epithelial stem cells in structures called the Lieberkühn crypts continuously divide and differentiate to replace damaged or sloughed‐off cells. Goblet cells are specialized epithelial cells that play a key role in producing mucus to protect and maintain the gut epithelial integrity and the commensal microbiota.^[^ ^54^ ^]^ To gain insights into the role of PBMT‐mediated alleviation of disease severity induced by lactic acid, we conducted periodic acid‐schiff (PAS) and BrdU staining of the intestine (Figure S3c, Supporting Information) and caecum (Figure 5e). Abdominal light exposure significantly increased crypt length (>120 µm) in the duodenum (*P* < 0.0001), jejunum (*P* < 0.0001), ileum (*P* = 0.0310), and caecum (*P* = 0.0015) as compared to disrupted crypts <120 µm in sham‐treated controls (Figure 5f). The intestinal crypts, which are invaginations of the epithelium, play a crucial role in maintaining gut homeostasis by harboring the intestinal stem cells responsible for replenishing the epithelial lining.^[^ ^55^ ^]^ In alignment with this, a significantly high number of PAS‐positive goblet cells were found in the duodenum (*P* < 0.0001), jejunum (*P* = 0.0001), ileum (*P* < 0.0001), and caecum (*P* = 0.0003) in LLL‐treated mice compared to the sham treatment (Figure 5g). PAS‐positive cells in the intestine signify the presence of goblet cells, which play a vital role in maintaining gut homeostasis by secreting mucus and forming a protective barrier shielding intestinal epithelium from harmful substances and pathogens. This mucus layer also facilitates nutrient absorption and supports the balance of the gut microbiota.^[^ ^56^ ^]^ Similarly, BrdU‐positive cells were observed in the duodenum (*P* = 0.0015), jejunum (*P* = 0.0002), and caecum (*P* = 0.0015) at a number significantly higher in LLL‐treated mice than in the sham treatment controls, despite no statistical difference seen in the ileum (Figure 5h). Increased cell proliferation and turnover rate of gut epithelium in LLL‐treated mice provide insights into intestinal cell renewal and maintenance dynamics, which help to maintain gut homeostasis even after repeated lactate injections. These data support the direct impact of LLL on the repair and maintenance of the gut epithelial barrier integrity.

## 3 Discussion

Brief abdominal PBMT, either alone or combined with thigh application, extends endurance, lowers lactate, and preserves muscle mitochondria under anaerobic exercises. Mechanistically, PBMT i) maintains epithelial integrity and renewal, as shown by increased crypt length, goblet cell density, and epithelial proliferation, and ii) directly promotes the growth of beneficial anaerobes in acidic, oxygen‐limited gut environments. The dual effects act both independently and synergistically, establishing a virtuous cycle that reinforces gut integrity and elevates key metabolites systemically (butyrate, spermidine, L‐carnitine, acetyl‐L‐carnitine). These metabolites fuel mitochondrial bioenergetics in distant muscles, substantially enhancing physical performance. While PBMT's role in preserving gut barrier has been studied, this is the first demonstration of its ability to directly promote the growth of beneficial anaerobes in acidic, oxygen‐limited gut environments. Unlike conventional probiotics and fecal microbiota transplantation (FMT), which often struggle with poor engraftment in dysbiosis environments, PBMT is host‐centered, noninvasive, and durable. By improving the gut niche and facilitating endogenous consortia without reliance on bacterial engraftment, PBMT avoids the risks of FMT, including pathogen transmission and limited durability. Given that dysbiosis and lactic acidosis are hallmarks of metabolic syndrome, inflammatory bowel disease (IBD), cancers, and neuroinflammatory disorders,^[^ ^50^ ^]^ PBMT can potentially be a versatile therapeutic strategy by reshaping the microbiome, reducing lactate accumulation, and enhancing mitochondrial efficiency.^[^ ^7^ ^]^

PBMT's translation potentials align with prior studies demonstrating its capacity to enhance mitochondrial biogenesis.^[^ ^8^, ^57^, ^58^ ^]^ LLL has been reported to stimulate mitochondrial respiratory chain (MRC) activity by directly activating complexes I, III, and IV, converting light energy into bioenergy in the form of ATP within the inner mitochondrial membrane.^[^ ^59^, ^60^ ^]^ Although bacteria lack mitochondria, they possess analogous components such as ATP synthase (complex V), a proton‐motive force, and an electron transport chain within their inner membrane. This similarity is not surprising, given the evolutionary origin of mitochondria from ancestral bacteria. Consequently, some bacteria, particularly facultative anaerobes in the gut, may respond to light similarly to mitochondria, utilizing light energy to generate ATP and support their growth. At present, the mechanisms by which 980 nm light promotes anaerobic bacterial growth remain unknown. In mammalian cells, however, the 980 nm laser has been shown to augment mitochondrial function by activating calcium channels in the cell membrane.^[^ ^61^, ^62^ ^]^ An influx of calcium ions can, in turn, stimulate mitochondrial activity, promoting ATP production and overall cellular energy metabolism.^[^ ^63^ ^]^ These effects are particularly pronounced in tissues with high energy demands, such as platelets, muscles, and the nervous system.^[^ ^64^, ^65^ ^]^ Strikingly, we found that ≈30‐fold lower fluence of 980 nm light (<0.1 J cm^−2^) enhanced mitochondrial function at levels comparable to 3 J cm^−2^ of 810 nm.^[^ ^8^, ^65^, ^66^ ^]^ This suggests that 980 nm light may hold stronger translational potential than 810 nm, as delivering 0.1 J cm^−2^ is more feasible and safer than 3 J cm^−2^ with respect to heat management. Furthermore, multiple clinical studies have reported PBMT‐mediated enhancement of mitochondrial function, reduction of oxidative stress, and improved tissue repair and recovery.^[^ ^67^, ^68^, ^69^, ^70^ ^]^ These human studies support its potential as a safe, adjunctive therapy to optimize cellular function and systemic energy balance.

Our microbiome analyses confirmed that PBMT selectively enriched beneficial anaerobic bacteria both in vivo and ex vivo, particularly *Firmicutes*, *Verrucomicrobiota*, and *Actinobacteria*, which are key SCFA producers. The restoration of butyrate‐producing genera, such as *Anaerostipes, Roseburia*, and *Butyrivibrio*, is particularly relevant given butyrate's role in gut epithelial protection, inflammation suppression, and mitochondrial energy metabolism.^[^ ^71^ ^]^ Furthermore, PBMT enhanced spermidine, L‐carnitine, and acetyl‐L‐carnitine levels, all of which contribute to mitochondrial function and metabolic resilience.^[^ ^72^ ^]^ These findings highlight PBMT's ability to re‐establish a healthy microbiota with which epithelial repair and energy metabolism can be further facilitated in the gut, providing a framework to identify specific dysbiosis states in humans that may be rectified by PBMT. Limitations of the work include the need for human dose translation and deeper mechanistic dissection. Nonetheless, convergent in vivo and ex vivo data support direct microbe responsiveness to 980 nm PBMT. Another limitation lies in the inherent differences in gut microbiota composition, which can vary drastically across institutions, housing conditions, or dietary backgrounds. While such variability was minimized by using co‐housed animals under controlled conditions, we recognize that microbial responses may differ in human populations with distinct diets and environments

## 4 Conclusion

This study demonstrates for the first time that non‐invasive abdominal PBMT significantly enhances physical performance by preserving gut epithelial integrity, promoting the growth of beneficial microbiota, and increasing the production of metabolites such as butyrate, spermidine, carnitine, and acetyl‐L‐carnitine. These metabolites support mitochondrial energy metabolism, muscle endurance, and epithelial repair under anaerobic stress. Our findings establish PBMT as a unique therapeutic strategy that integrates gut microbiota modulation with systemic metabolic benefits. The translational implications are broad, spanning from athletic performance, military endurance, to various clinical conditions characterized by muscle atrophy, mitochondrial dysfunction, or gut dysbiosis. By leveraging a novel gut‐muscle axis mechanism, our work opens new avenues for non‐invasive, home‐based PBMT interventions to promote health and performance.

## 5 Experimental Section

### Animals

BALB/c mice were obtained from Jackson Laboratories and housed in pathogen‐free animal facilities at Massachusetts General Hospital (MGH), under constant environmental conditions (temperature 22–24 °C, a 12 h light/dark cycle, and 50 ± 10% humidity). The mice had free access to food and water. All animal‐related procedures were conducted in accordance with institutional, hospital, and NIH guidelines and were reviewed and approved by the MGH Institutional Animal Care and Use Committee (Approval Number 2020N000083). The mice were allowed to acclimate for one week prior to the experiments.

### Experimental Design and Sample Collection

To deplete the densely populated gut microbiota, mice were administered an oral antibiotic cocktail daily for 15 days. The cocktail consisted of amoxicillin (200 mg kg^−1^), metronidazole (200 mg kg^−1^), streptomycin (200 mg kg^−1^), and vancomycin (100 mg kg^−1^). The mice were hair removed on the upper portion of two rear legs and abdomen and randomly assigned to the following groups: 1) no exercise control; 2) sham treatment control with exercise; 3) exercise with LLLT on two legs (iL); 4) exercise with LLLT applied on the abdomen (iA); and 5) exercise with LLLT on both legs and abdomen exercise (iL + A). The body weight was recorded weekly throughout the study. On the last day of the study, hind limb muscle (soleus, gastrocnemius), caecum, feces, and serum samples were collected after sacrifice. The serum samples were used for carnitine, acylcarnitine, and butyrate measurement through liquid chromatography‐mass spectrometry quadrupole time‐of‐flight (LC‐MS Q‐TOF), while the fecal samples were used for butyrate measurement through gas chromatography‐mass spectrometry quadrupole time‐of‐flight (GC‐MS Q‐TOF). Qualitative and quantitative analyses of mitochondria from the soleus and gastrocnemius muscles were performed using transmission electron microscopy (TEM) and flow cytometry after mitochondrial staining. Hematoxylin and eosin (H&E) and bromodeoxyuridine (BrdU) staining were conducted on the intestine (duodenum, jejunum, and ileum) and caecum. The fecal DNA was used for 16S rRNA gene analysis. In the acidosis model, the dose of sodium L‐lactic acid in mice was first optimized to achieve similar lactate levels as seen during high‐intensity exercise under anaerobic conditions. Mice were intraperitoneally administered sodium L‐lactic acid at a dose of 1, 2, 3, or 4 mg kg^−1^, respectively, followed by measuring the body weight and lactate levels at the indicated days. After dose optimization, BALB/c mice at six‐to‐eight‐weeks of age were treated with the antibiotic cocktail as above and then intraperitoneally administered sodium L‐lactic acid at a dose of 3 g kg^−1^ on days 1 and 7. The mice were treated with either sham light or illumination on the abdomen (iAbdomen). The body weight and disease activity scores were monitored daily. On day 10, the mice were dissected, and the intestine (duodenum, jejunum, and ileum) and caecum were collected for histological analysis, including H&E staining, BrdU staining, and PAS.

### Low‐Level Light Therapy (LLLT)

A 980 nm laser (OsTech, Berlin, Germany) at a fluence of 0.1 J cm^−2^ was applied on shaved areas of the hind limb (iL) or abdomen (iA), either alone or both (iL+A). The laser fluence was achieved by exposing the designated area to 170 mW cm^−2^ for 60 s or indicated times as measured using a Handheld Laser Power & Energy Meter (ThorLabs GmbH). The 980 nm laser beam was transmitted through a 6 mm diameter optical fiber.

### Anaerobic Exercise on the Treadmill

Prior to the exercise training with the treadmill, a one‐week adaptation period was implemented for all mice, with the initial settings of the shock grid at 0.9 mA (Maze Engineers Automated Treadmill, Skokie, IL, USA), after which a progressive exercise training regimen was carried out five days per week following a standard or published procedure.^[^ ^73^ ^]^ The speed and intensity of the treadmill were gradually increased from 5 to 25 m min^−1^. After acclimatization to the training, mice underwent a graded maximal exhaustive test. Exhaustion was defined by the occurrence of any of the following criteria: 1) remaining on the electric grid for 10 consecutive seconds, 2) spending more than 50% of the time on the grid, and/or 3) showing a lack of motivation requiring manual prodding. Once any criterion was met, mice were immediately removed from the treadmill, and exhaustion time was recorded. The graded maximal exhaustive test began with 5 m min^−1^ for 5 min, followed by 15 m min^−1^ for 10 min, 20 m min^−1^ for 15 min, and finally 25 m min^−1^ until exhaustion. Additionally, exhaustion was confirmed if a mouse received more than 20 shocks within a 5‐min interval during the final stage. Total running distance (in meters) and exhaustion time (in minutes) were recorded. To simulate an anaerobic environment for high‐intensity exercise, the treadmill was adjusted to a 10° incline.

### Blood Sampling and Lactate Measurements

Blood lactate concentration was measured with the Lactate Plus handheld blood lactate meter (Nova Biomedical, Waltham, MA, US) in mice immediately after exercise. As adapted from lactate measurement protocols, one drop of blood was collected via tail vein puncture onto a disposable strip for lactate analysis. Vein puncture was preferred due to its accessibility in mice. Additionally, a recent study in humans suggested that arterial and venous blood lactate could be used interchangeably as long as the lactate measurements were taken within 10 min after the start of the exercise bout.

### Serum L‐Carnitine, Acetyl‐L‐Carnitine, and Spermidine Quantification

The serum sample was dissolved in 100 µL ice‐cold ethanol under ultrasonication at 4 °C, filtered through a 0.2 µm membrane in a Whatman syringe, and then transferred into LC‐MS vials. LCMS conditions were set as previously described.^[^ ^74^ ^]^ Analytes were measured in scheduled multiple reaction monitoring (MRM) and Product ion (PI). A standard curve was established with different concentrations of a standard mixture containing acetyl‐L‐carnitine and L‐carnitine standards.^[^ ^75^, ^76^ ^]^ Spermidine was also quantified similarly. A ten‐point calibration was performed to construct calibration curves, and fitted by linear regression.^[^ ^77^ ^]^

### Fecal Butyrate Quantification

The butyrate in the plasma and feces was detected by gas chromatography mass spectrometry (GC) as previously described.^[^ ^78^ ^]^ Briefly, the plasma (100 µL) or feces (150–200 mg) was mixed with 1 mL ethanol and molecular sieves size 9Å and vortexed at room temperature for 10 min. Then, the samples were treated with 150 µL of 50% H_2_SO_4_ (w/w), fully vortexed, and kept at around 80 °C overnight, to which some sodium carbonate 200 mg and 1 mL of hexane were added the next day, after cooling the samples. The supernatant was obtained by centrifugation (8000 rpm, 5 min, 4 °C), filtered through a silica‐filled column tube, and transferred to a clear GC vial. The concentration of butyrate was analyzed using GC‐2014C (Agilent), fitted with a DB‐FFAP capillary column (30 m × 0.25 µm × 0.25 mm) (Agilent Technologies, Wilmington, DE, USA). A standard curve was established with different concentrations of a standard mixture containing ethyl butyrate (Fisher Scientific). Peaks were integrated by using the Agilent qualitative analysis solution software. Butyrate concentration is expressed as ng mL^−1^ in plasma or µg g^−1^ in feces.^[^ ^78^ ^]^

### Transmission Electron Microscopy (TEM)

Muscle tissues were fixed in Karnovsky fixative (K2 buffer) (Electron Microscopy Sciences, EMS, Hatfield, PA), consisting of 2.5% glutaraldehyde and 2% paraformaldehyde in 0.1M  sodium cacodylate buffer (wash buffer) at 4 °C for up to 24 h. The tissues were post‐fixed with 1% OsO4 plus 1.5% potassium ferrocyanide (EMS) and then subjected to standard processes for TEM staining and embedding in Epon t812 (Tousimis, Rockville, MD). Semi‐thin sections (0.5 µm) were cut and stained with Toluidine Blue. Ultrathin sections (80 nm) were cut using a Reichert‐Jung Ultracut E microtome (Vienna, Austria), collected on uncoated 100‐mesh copper grids or slots, and examined on a Philips CM‐10 Transmission Electron Microscope (Eindhoven, The Netherlands). Digital TEM images were taken by AMT‐XR41M 4.0 Megapixel Cooled sCMOS camera (Advanced Microscopy Techniques, Woburn, MA). Mitochondrial analysis was performed on 14000× cross‐sections of the gastrocnemius and soleus muscles. ImageJ was used to calculate cristae size and the number, blinded to study groups.

### Cell Isolation and Flow Cytometry

Single cells were isolated from the gastrocnemius and soleus muscles after the tissues were minced and digested with collagenase IV (1 unit mL^−1^), DNase I (0.1 mg mL^−1^), and RPMI for 45 min. The cell suspension was centrifuged at 500 g for 5 min, and the pellet was incubated for another 45 min in a fresh digestion buffer. The cell suspension was passed 20 times through a 10‐mL pipette to release cells, centrifuged at 50 g for 5 min to remove large debris, and passed through a 70‐mm cell strainer. The mitochondrial mass was assayed by flow cytometry using Mito Tracker Deep Red FM per the manufacturer's instructions (Molecular Probes, Invitrogen, Waltham, MA). The cells were analyzed by flow cytometry after intracellular staining (FACSAria, BD Bioscience, San Jose, CA).

### BrdU Staining

Mice to be tested were i.p. injected with 1 mL (1 mg mL^−1^) 5‐bromo2′‐deoxyuridine (BrdU) and sacrificed 2 h later. Colons and intestines were removed, fixed, and sectioned as above. BrdU staining was performed using a Zymed BrdU labeling kit (BD Bioscience, USA) per the manufacturer's instructions. BrdU‐positive cells were counted in 5–10 crypts in each group, and the average number of positive cells per crypt was presented.

### Histological Analysis

The duodenum, jejunum, ileum, and caecum were dissected from the gastrointestinal tract. The specimens were immersed in 10% buffered formalin for 48–72 h to fix them. Subsequently, they were dehydrated, cleared, and embedded in paraffin. Serial sections with a thickness of 5 µm were obtained from the embedded tissues. These sections were then deparaffinized, rehydrated, and subjected to staining using H&E, PAS, and BrdU techniques. Images were captured using a NanoZoomer system to analyze the stained sections, and NDP view software (Hamamatsu, Bridgewater, NJ) was utilized. The software allowed for measurements of crypt length in the caecum and villi length in the small intestine.

### 16s rRNA Gene Sequencing

Before the mice were sacrificed, the feces were collected quickly in a sterile environment. According to the manufacturer's instructions, the PureLink Genomic DNA Mini Kit was used to isolate fecal DNA. The DNA samples (10 ng) were amplified by qPCR using PCR primers specific for various phyla (**Table** **3**) as previously described.^[^ ^79^ ^]^ The specific primers were selected to amplify the V3–V4 region of the 16s rRNA gene. The samples were sequenced on the Illumina Miseq platform (Illumina, San Diego, CA, USA) using a 2 × 250 bp V2 sequencing kit. Sequences were sequentially depleted of barcodes, primers, short sequences < 200 bp, ambiguous base calls, and homopolymers exceeding 6 bp. Then, sequences were denoised and chimeras were removed. Operational taxonomic units (OTUs) were defined after removing singleton sequences, clustering at 3% divergence (97% similarity). OTUs were taxonomically classified using BLASTn against a curated GreenGenes/RDP/NCBI‐derived database.

**Table 3:** 16s rRNA Genes and primer sequences used in qPCR assays.

Genes	Forward (5′‐3′)	Reverse (5′‐3′)
Bacteroidetes	GTTTAATTCGATGATACGCGAG	TTAASCCGACACCTCACGG
Firmicutes	GGAGYATGTGGTTTAATTCGAAGCA	AGCTGACGACAACCATGCAC
Actinobacteria	TGTAGCGGTGGAATGCGC	AATTAAGCCACATGCTCCGCT
Deferribacteres	CTATTTCCAGTTGCTAACGG	GAGHTGCTTCCCTCTGATTATG
Verrucomicrobia	TCAKGTCAGTATGGCCCTTAT	CAGTTTTYAGGATTTCCTCCGCC
Tenericutes	ATGTGTAGCGGTAAAATGCGTAA	CMTACTTGCGTACGTACTACT
Delta‐proteobacteria	GCTAACGCATTAAGTRYCCCG	GCCATGCRGCACCTGTCT

### Ex Vivo Bacterial Culture

Fresh fecal samples were collected from healthy naïve mice and immediately suspended in pre‐warmed, phenol red‐free RPMI 1640 medium at a ratio of 1 g of feces per 9 mL of medium. The suspension underwent thorough vortexing and subsequent centrifugation at 4500 g for 5 min to obtain supernatant. The supernatant was mixed with low pH (5.5 and 6.5) RPMI media adjusted using hydrochloric acid (HCl). Before spreading on the red blood agar (RBA) plates, the treated groups were exposed to 980 nm LED light at a dose of 0.1 J cm^−2^ in the tube. After the fecal bacteria were spread on the plates, they were exposed to the same light treatment. This light treatment was repeated once a day for the next two days, making a total of four treatments. The RBA plates were then incubated anaerobically at 37 °C for 72 h (Figure 4a). Bacterial samples were collected on days 1 and 3 for DNA isolation as above for qPCR analysis. The control group received sham light treatment.

### Statistical Analysis and Reproducibility

Results are presented as means ± S.D./SEM, as specified in the figure legends, with individual animal data displayed in the bar graphs. Gut microbiota data were analyzed as described in the section on gut microbiota profiling. The other data were analyzed by one‐way ANOVA, followed by the Bonferroni post hoc test, to determine statistical significance. A *p* < 0.05 was considered significant based on two‐tailed hypothesis testing. All statistical analyses were performed using GraphPad Prism version 10.2. All experiments were repeated at least once with similar results unless otherwise specified.

### Ethics Declarations‐Ethics Approval and Consent to Participate

All animal‐related procedures were conducted in accordance with the institutional, hospital, and NIH guidelines and were reviewed and approved by the Massachusetts General Hospital Institutional Animal Care and Use Committee, Boston, USA (Approval Number 2020N000083).

## Conflict of Interest

The authors declare no conflicts of interest.

## Author Contributions

M.X.W. and P.U. conceived the study and designed the research. P.U. conducted experiments. B.B. contributed to the development of LC‐MS and GC‐MS methods of analysis with P.U., and A.B. contributed to the flow cytometry experiment. P.U. and M.X.W. wrote the paper. M.X.W. supervised the project and secured the funding for the study.

## Supporting information



Supporting Information

## Data Availability

All data are available in the main text or supplementary materials. The raw sequencing data generated in this study have been deposited in the NCBI Sequence Read Archive (SRA) under BioProject accession number PRJNA1270724.
