# Supplementary material for: Photobiomodulation Strengthens Muscles via Its Dual Functions in Gut Microbiota
Source: Adv Sci (Weinh). 2025 Sep 23;12(46):e11582. doi: 10.1002/advs.202511582 (PMC12697884; doi:10.1002/advs.202511582)
Supplement: Supplementary file 1 — Supporting Information [file ADVS-12-e11582-s001.pdf]

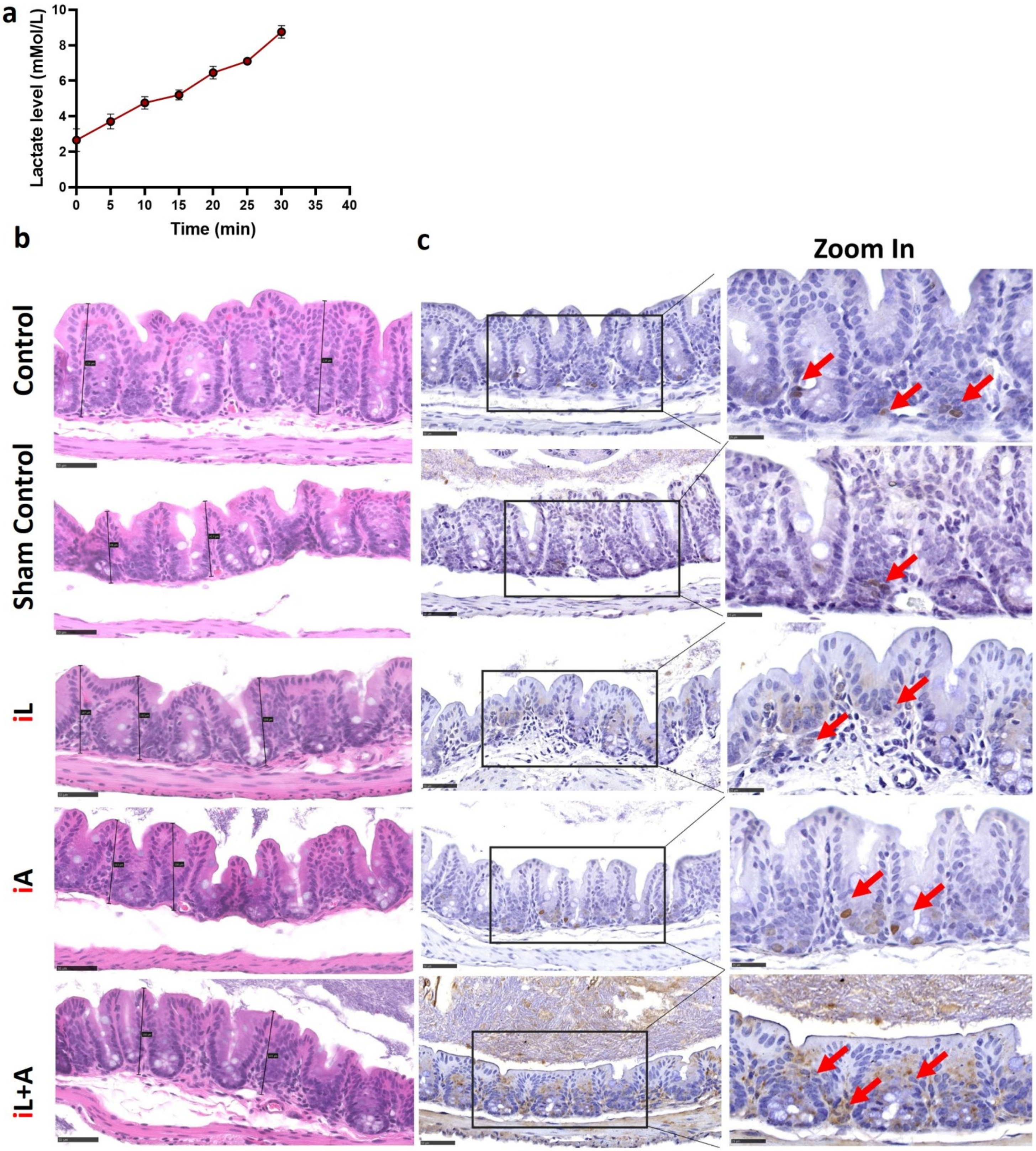

Supplementary Figure S1. Lactate levels during anaerobic exercise and effects of abdominal LLLT on gut integrity. **(a)** Lactate level (mMol/L) increases during high-intensity anaerobic exercise on a treadmill. Data are presented as mean ± SEM (n =8). **(b)** Representative and enlarged images of H &E-stained caecum corresponding to Fig 2a. Scale bar, 50 µm. **(c)** Representative images of BrdU-stained caecum with zoom in, corresponding to Fig. 2b. Red arrows indicate BrdU-positive cells. Scale bar, 50 (left) & 25 µm (right).

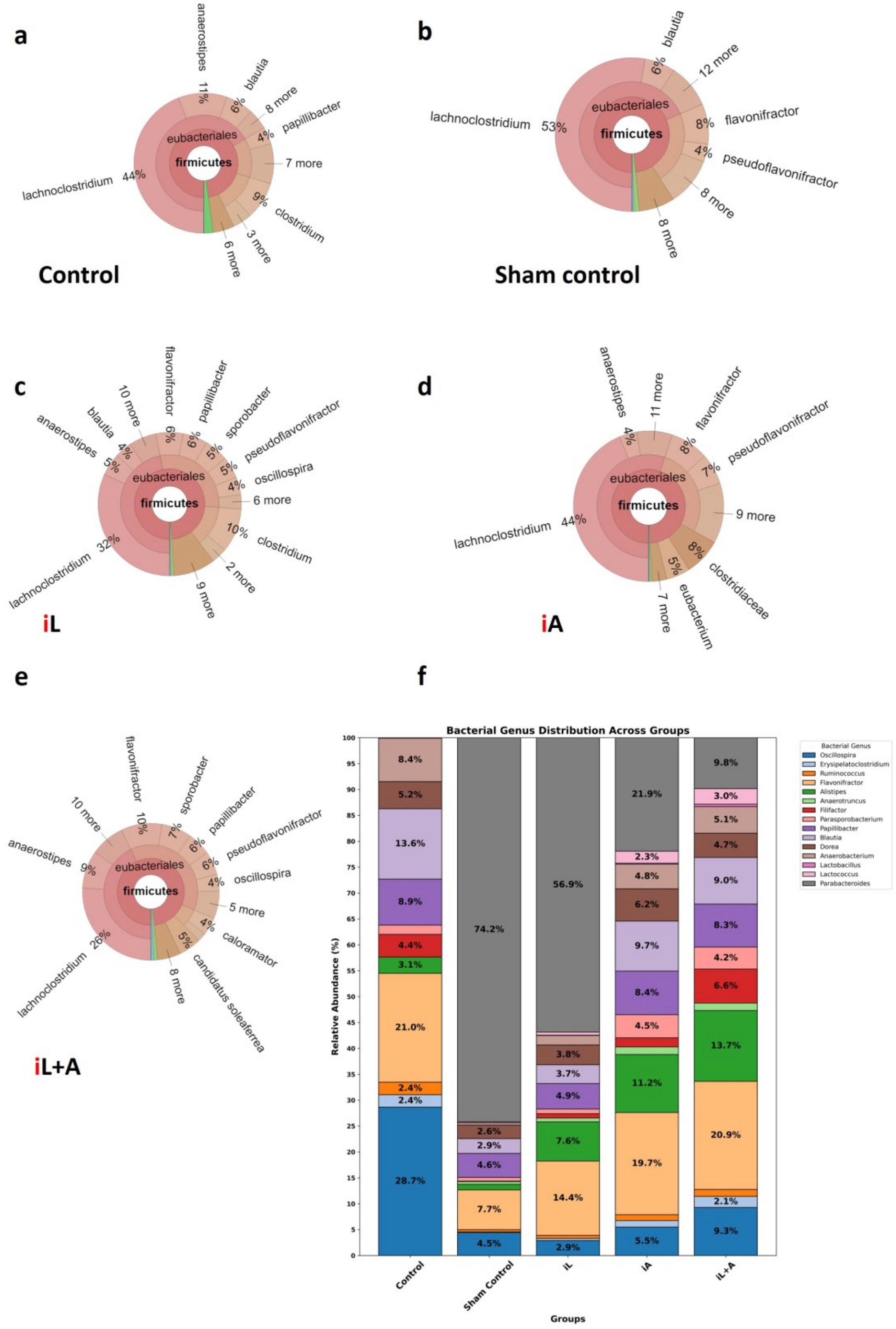

Supplementary Figure S2: PBMT restores the firmicutes obligate/facultative anaerobic bacteria. Related to Fig. 2 (l–p). Representative images from the Krona analysis chart software displaying taxonomy at the genus level within the *Firmicutes* phylum, Control **(a)**, Sham control **(b)**, iL **(c)**, iA **(d)**, and iL+A **(e)**. **(f)** A stacked bar graph illustrating the percentage of total relative abundance of bacterial genera across the groups of obligate and facultative anaerobic bacteria within the *Firmicutes*, *Bacteroidetes*, and *Tenericutes* phyla, particularly those associated with muscle mitochondria and metabolite production.

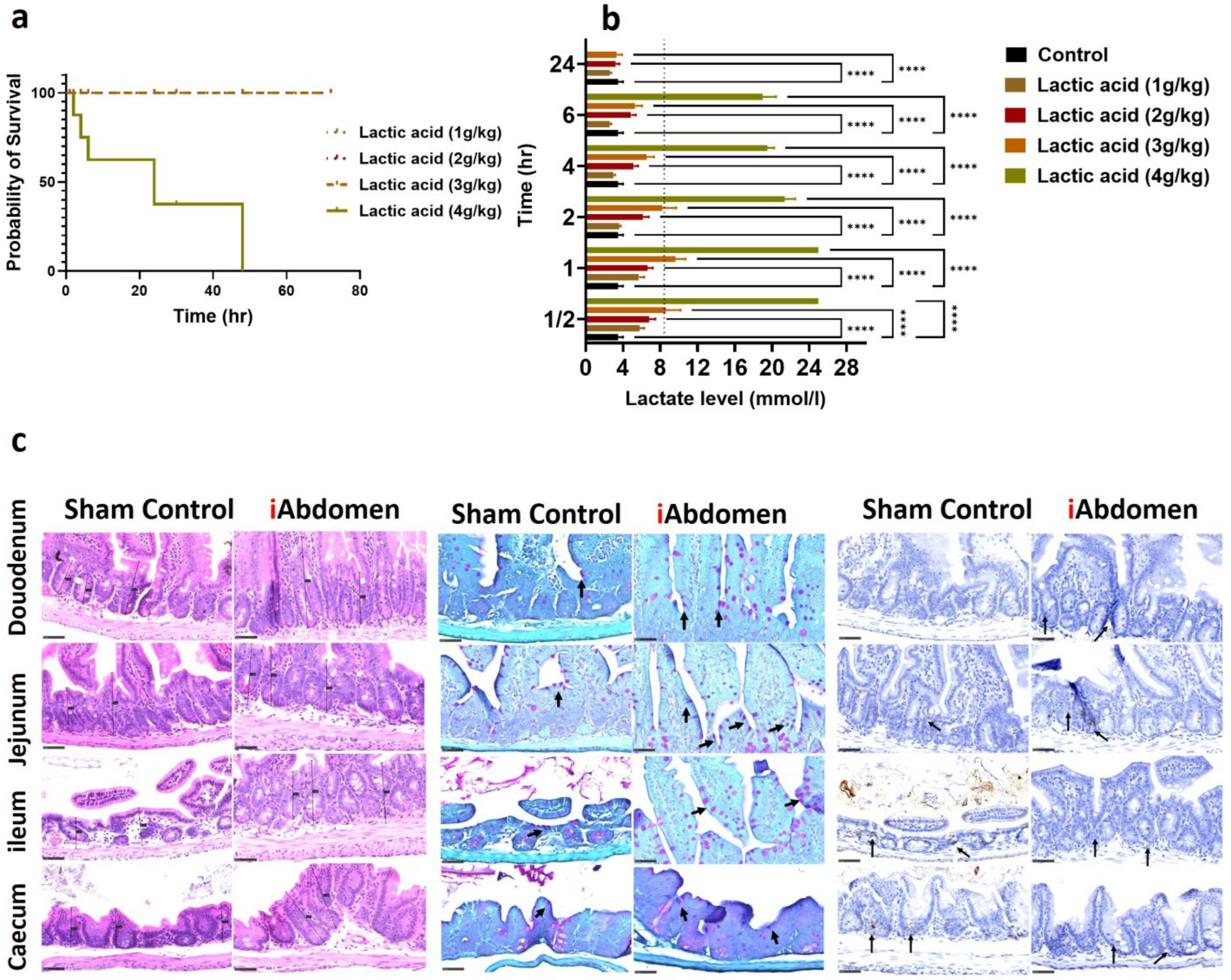

Supplementary Figure S3: **Abdominal LLLT reshapes the gut epithelial wall under lactate-mediated acidosis. Related to Fig 5, (a)** Kaplan Meier survival analysis of Lactic acid-induced gut dysbiosis; (n=10). **(b)** Lactate level (mmol/L): The dashed line showed the highest level of lactate present in mice during intense anaerobic exercise (n=10). **(c)** Representative image of the duodenum, jejunum, ileum, and caecum stained with H&E, PAS, and BrdU. Scale bar, 50μm.
